# Supplementary material for: Synthetic cells with self-activating optogenetic proteins communicate with natural cells
Source: Nat Commun. 2022 Apr 28;13:2328. doi: 10.1038/s41467-022-29871-8 (PMC9050678; doi:10.1038/s41467-022-29871-8)

Supplementary Information

Synthetic Cells with Self-Activating Optogenetic Proteins Communicate with Natural Cells

Omer Adir[1,2], Mia R. Albalak[1,3], Ravit Abel[1,2], Lucien E. Weiss[4,5,6], Gal Chen[1,3], Amit Gruber[7], Oskar Staufer[8,9,10], Yaniv Kurman[11], Ido Kaminer[11], Jeny Shklover[1], Janna Shainsky-Roitman[1], Ilia Platzman[8,9], Lior Gepstein[7,12], Yoav Shechtman[4,5], Benjamin A. Horwitz[13], Avi Schroeder[1*]

[1] The Louis Family Laboratory for Targeted Drug Delivery and Personalized Medicine Technologies, Department of Chemical Engineering, Technion, Haifa, Israel

[2] The Norman Seiden Multidisciplinary Program for Nanoscience and Nanotechnology, Technion, Haifa, Israel

[3] The Interdisciplinary Program for Biotechnology, Technion, Haifa, Israel

[4] Department of Biomedical Engineering, Technion, Haifa, Israel

[5] Lorry I. Lokey Interdisciplinary Center for Life Sciences and Engineering, Technion, Haifa, Israel

[6] Department of Engineering Physics, Polytechnique Montréal, Montréal, Canada

[7] Sohnis Research Laboratory for Cardiac Electrophysiology and Regenerative Medicine, the Rappaport Faculty of Medicine and Research Institute, Technion, Haifa, Israel

[8] Department for Cellular Biophysics, Max Planck Institute for Medical Research, Heidelberg, Germany

[9] Institute for Molecular Systems Engineering (IMSE), Heidelberg University, Heidelberg, Germany

[10] Max Planck School Matter to Life, Heidelberg, Germany

[11] The Andrew and Erna Viterbi Faculty of Electrical and Computer Engineering, Technion, Haifa, Israel

[12] Cardiology Department, Rambam Health Care Campus, Haifa, Israel

[13] Faculty of Biology, Technion - Israel Institute of Technology, Haifa, Israel

* **Corresponding author**. email: avids@technion.ac.il

Supplementary Table of Contents

Supplementary Notes 4

Plasmids and hosts 4

Protein expression and purification 4

Supplementary Tables 6

Supplementary Table 1. Primers used for plasmid design. 6

Supplementary Table 2. Synthetic cells' inner solution original composition – before adaptations for production and correct folding of *Gaussia* luciferase. 7

Supplementary Table 3. Synthetic cells' inner solution composition– after adaptations for production and correct folding of *Gaussia* luciferase. 7

Supplementary Table 4. Synthetic cells' outer solution composition. 8

Supplementary Figures 9

Supplementary Figure 1. Size distribution analysis of 100-nm POPC liposomes using dynamic light scattering 9

Supplementary Figure 2. The effect of cholesterol addition to the liposomal membrane on blue light absorbance in POPC liposomes.. 9

Supplementary Figure 3. CryoSEM images distinguishing between synthetic cells and residue oil droplets from the preparation method 10

Supplementary Figure 4. The absorbance spectrum of 100 nm liposomes composed of different phospholipids. 11

Supplementary Figure 5. The effect of different concentrations of oxidized and reduced glutathione on *Gaussia* luciferase production in cell free reactions. 11

Supplementary Figure 6. SDS-Page gel with coomassie blue staining of the purified fraction of disulfide bond isomerase C (DsbC). 12

Supplementary Figure 7. Comparison of light emission levels in *Gaussia* luciferase-expressing cell-free protein expression (CFPS) reactions and synthetic cells between self-prepared and commercial internal solutions. 12

Supplementary Figure 8. The effect of incubation temperatures on *Gaussia* luciferase expression in SCs 13

Supplementary Figure 9. Light emission from *Gaussia* luciferase-expressing synthetic cells with variable concentrations of coelenterazine ranging from 10 nM to 100 µM. 13

Supplementary Figure 10. Induction of photoconidiation in *Trichoderma atroviride* colonies after exposure to blue light 14

Supplementary Figure 11. SDS-Page gel with coomassie blue staining of the purified *Gaussia* luciferase (Gluc) – EL222. 14

Supplementary Figure 12. The effect of coelenterazine addition in different concentrations on the yield of GFP production in cell free protein synthesis reactions. 15

Supplementary Figure 13. SDS-Page gel with coomassie blue staining of the his-MBP-mRFP-sspB-Nano after TEV restriction, before and after purification of mRFP-sspB-Nano. 15

Supplementary Figure 14. Recruitment of RFP-sspB-Nano to synthetic cells with iLID functionalized membrane using a CFPS reaction expressing *Gaussia* luciferase supplemented with 100 μM coelenterazine. 16

Supplementary Figure 15. SDS-Page gel with coomassie blue staining of the purified Gluc– iLID...16

Supplementary Figure 16. Recruitment of RFP-sspB-Nano to synthetic cells functionalized with iLID or Gluc-iLID after 4 minutes of exposure to external 488 nm laser light or after four additions of 0.2 nmol of coelenterazine.......................17

Supplementary Notes

Plasmids and hosts

The DNA sequences of all the proteins used in this study are listed in supplementary table 1. *E. coli* DH5α and TOP10 strains were used for cloning and plasmid purification. DNA sequences of the engineered plasmids were confirmed by sequencing. A plasmid expressing *Renilla* luciferase (Rluc) under the T7 promoter was obtained from the S30-T7 high yield protein expression system kit, purchased from Promega (USA). *Gaussia* luciferase (Gluc)-expressing plasmid was generously provided Prof. James Swartz (department of chemical engineering, Stanford university). Plasmids expressing DsbC (Plasmid #38152), EL222 (Plasmid #113108), iLID (Plasmid #60408), sspB-Nano (Plasmid #60409), and pBLind RFP (Plasmid #113109) were purchased from Addgene (USA). PCR was used to add a C-terminal his tag to the DsbC protein and insert it to a pET28a vector. C-terminal his tag was also added to the EL222 sequence that was isolated and inserted into a pET28a vector. Hifi DNA assembly (NEB, USA) was used to produce Gluc-EL222-his and his-Gluc-iLID sequences, each inserted to a pet28a vector (deposited in Addgene, IDs #172097 and #172096 respectively). mRFP1 sequence was inserted between the MBP and sspB-Nano reading frames to generate a his-MBP-RFP-sspB-Nano vector. A plasmid expressing Rluc under the pBLind promoter was produced by replacing the RFP sequence in the original vector with the Rluc sequence from the Rluc expressing plasmid. A complete list of the primers used for plasmid design is available in Supplementary Table 1.

Protein expression and purification

BL21(DE3) *E. coli* (NEB) were used for the expression of DsbC-his, EL222-his, iLID and his-maltose binding protein (MBP)-mRFP1-sspB-Nano. Expression of Gluc-EL222-his and his-Gluc-iLID was performed in SHuffle T7 *E. coli*. A 5 ml Luria-broth starter culture for each protein was incubated overnight at 37 ºC and 250 rpm with the compatible antibiotics (ampicillin at 100 μg ml^{-1} or kanamycin at 25 μg ml^{-1}). The starter was then transferred to 500 ml of Terrific-broth supplemented with antibiotics in the same concentration and grown at 37 ºC and 250 rpm to optical density (OD) of 0.5, when they were induced with 500 μM of Isopropyl β-D-1-thiogalactopyranoside (IPTG). DsbC-his and EL222-his were incubated at 37 ºC and 250 rpm following

induction until reaching an OD of ~4. His-iLID, his-MBP-mRFP1-sspB-Nano, Gluc-EL222-his and his-Gluc-iLID were grown at 16 ºC and 250 rpm until reaching similar OD values. Cells were harvested by centrifugation at 7,000 x g for 10 minutes at 4 °C and kept at -20 °C until the next step.

For protein purification, the pellet was resuspended in PBS (in the case of DsbC-his and EL222-his), 50 mM Tris, 300 mM NaCl, pH 7.4 (in the case of his-iLID and his-MBP-RFP-sspB-Nano) or 300 mM NaCl, 50 mM phosphate buffer, pH 8.0 (in the case of his-Gluc-iLID and Gluc-EL222-his). The cells were fractionated by two passes through an emulsiFlex-C3 high pressure homogenizer (Avestin, Germany) and the lysate was spun down two times for 15 minutes at 20,000 x g. The supernatant was passed through an AKTA purifier chromatography system (Cytiva, USA) using a HisTrap HP 5 ml column and eluted with elution buffer with similar composition to the loading buffer supplemented with 500 mM imidazole. The protein containing fractions were dialyzed in a 12-14 kD membrane (Spectrum Laboratories, USA) against their original resuspension buffer.

To remove the his-MBP domain from the mRFP1-SspB-Nano protein, the eluted MBP-RFP-sspB proteins were cut with TEV protease (NEB) using a digestion site between the MBP and the RPF sections. 300 µg of his-MBP-RFP-sspB-Nano were diluted to a total reaction volume of 880 µl. 20 µl of TEV Protease Reaction Buffer (10X) and 100 µl of TEV Protease were added, and incubated at 4°C overnight. 10 reactions samples were pooled together and passed through a Ni Sepharose 6 Fast Flow histidine-tagged protein purification resin (Cytiva). The flow-through containing the RFP-SspB-Nano protein, was collected and concentrated using Amicon ultra 15 kDa (Merck, USA). The proteins were dialyzed overnight in PBS.

Supplementary Tables

Primer	Sequence
DsbC forward primer	TAATACGACTCACTATAGGG
DsbC reverse primer	GGATCCTTAGTGGTGGTGGTGGTGGTGTTTACCGCTGGTCAT
EL222 forward primer	ATGCGCCATGGGTATGTTGGATATGGGACAAGATCGG
EL222 reverse primer	ATCGCGGATCCTTAGTGATGATGATGATGATGGATTCCGGCTTCGACGGCAA
mRFP1 forward primer	CAGTCGGATCCATGGCGAGTAGCGAAGACGT
mRFP1 reverse primer	AGCATCTGCAGAGCACCGGTGGAGTGACGA
pQE-sspB forward primer	AATGACTGCAGAGCTCCCCGAAACGCCCTAA
pQE-sspB reverse primer	GGAGCTGGATCCCTGAAAATACAGG
UV oligo forward primer	GGCGCAATCACGAATGAATA
UV oligo reverse primer	CTCAACCCTATCTCGGTCTA
iLID forward primer	GGATCCGGGGAGTTTCTGGC
iLID reverse primer	CAAGCTTGTCGACGGAGCTCGAATTCTTAAAAGTAATTTTCGTCGTTCGCTGC
Gluc forward primer	GTTTAACTTTAAGAAGGAGATATACATATGCATCACCATCACCATCACAAAC
Gluc reverse primer	GTCACCACCCGCGCCTTTGATC
Gluc-iLID-linker	TAAGATCAAAGGCGCGGGTGGTGACGGTGGTGGTGGTTCAGGTGGTGGTGGTTCAGGATCCGGGGAGTTTCTGGCAACCA
Gluc-EL222 forward primer	TTTTGTTTAACTTTAAGAAGGAGATATACATATGAAACC
Gluc-linker-rev	TCTTGTCCCATATCCAACATTGAACCACCACCACCTGAACCACCACCACCGTCACCACCCGCGCCTTT
Renilla luciferase forward primer	ATAGACATATGGCTTCCAAGGTGTACGA
Renilla luciferase reverse primer	GATTTGGATCCTTACTGCTCGTTCTTCAGCA

Supplementary Table 1. Primers used for plasmid design.

Reagent	final concentration	
Sucrose	200	mM
HEPES KOH (pH=8)	55	mM
Magnesium acetate	14	mM
Potassium acetate	50	mM
Ammonium acetate	155	mM
Polyethylene glycol 6000 (PEG)	3%	(w/v)
3-Phosphoglyceric acid (3-PGA)	40	mM
Amino acids - mixture I	2.5	mM
Amino acids - mixture II	2.5	mM
ATP	1.2	mM
GTP	1	mM
UTP	0.8	mM
IPTG	1	mM
S30-T7 lysate	34%	(v/v)
DNA	10	μg ml^{-1}

Supplementary Table 2. Synthetic cells' inner solution original composition – before adaptations for production and correct folding of *Gaussia* luciferase.

Reagent	final concentration	
Sucrose	200	mM
HEPES KOH (pH=8)	55	mM
Magnesium acetate	14	mM
Potassium acetate	50	mM
Ammonium acetate	155	mM
Polyethylene glycol 6000 (PEG)	3%	(w/v)
3-Phosphoglyceric acid (3-PGA)	40	mM
Amino acids - mixture I	2.5	mM
Amino acids - mixture II	2.5	mM
ATP	1.2	mM
GTP	1	mM
UTP	0.8	mM
IPTG	1	mM
S30-T7 lysate (**without DTT and β-mercaptoethanol**)	**57%**	**(v/v)**
DNA	10	μg ml^{-1}
**Disulfide bond isomerase C (DsbC)**	**75**	**μg ml^{-1}**
**Oxidized glutathione (GSSG)**	**4**	**mM**
**Reduced glutathione (GSH)**	**1**	**mM**

Supplementary Table 3. Synthetic cells' inner solution composition– after adaptations for production and correct folding of *Gaussia* luciferase. Modifications are highlighted in bold.

Reagent	final concentration	
glucose	200	mM
HEPES KOH (pH=8)	83.8	mM
Magnesium acetate	21.2	mM
Potassium acetate	75.8	mM
Ammonium acetate	236.4	mM
Polyethylene glycol 6000 (PEG)	4.5%	(w/v)
3-Phosphoglyceric acid (3-PGA)	60.6	mM
Amino acids - mixture I	3.8	mM
Amino acids - mixture II	3.8	mM
ATP	1.8	mM
GTP	1.5	mM
UTP	1.2	mM
IPTG	1.5	mM

Supplementary Table 4. Synthetic cells' outer solution composition.

Supplementary Figures

Supplementary Figure 1. Size distribution analysis of 100-nm POPC liposomes using dynamic light scattering (PDI = polydispersity index).

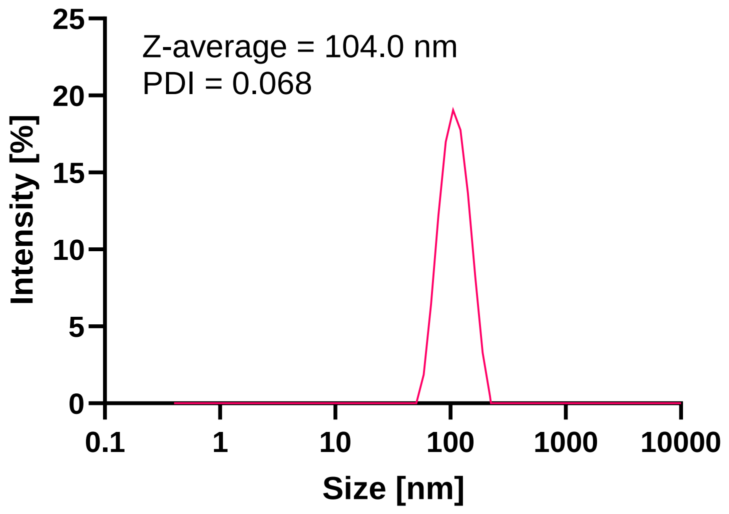

Supplementary Figure 2. The effect of cholesterol addition to the liposomal membrane on blue light absorbance in POPC liposomes. The absorbance of 100 nm liposomes with 40 mol% cholesterol or 0 mol% cholesterol at 480 nm was measured. Data is represented as the mean ± standard deviation (n=3 independent samples). Nested two-tailed t-test adjusted P value; *p=0.048.

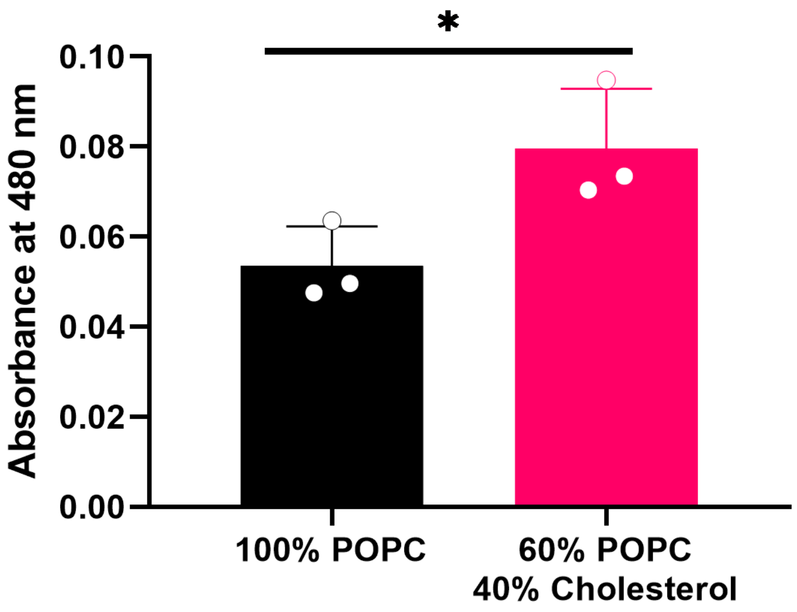

Supplementary Figure 3. CryoSEM images distinguishing between synthetic cells and residue oil droplets from the preparation method. Images (a) and (c) were captured with SE2 and InLens imaging detectors. Images (b) and (d) are corresponding images captured with the energy selective backscattered (ESB) detector, used for elemental analysis contrast between organic and aqueous phases. Dark domains in the ESB images represent the organic oil phase and light domains represent the aqueous phase. White arrows indicate synthetic cells and white arrowheads indicate oil droplets. This experiment was reproduced two times, ESB detector images were taken in one experiment.

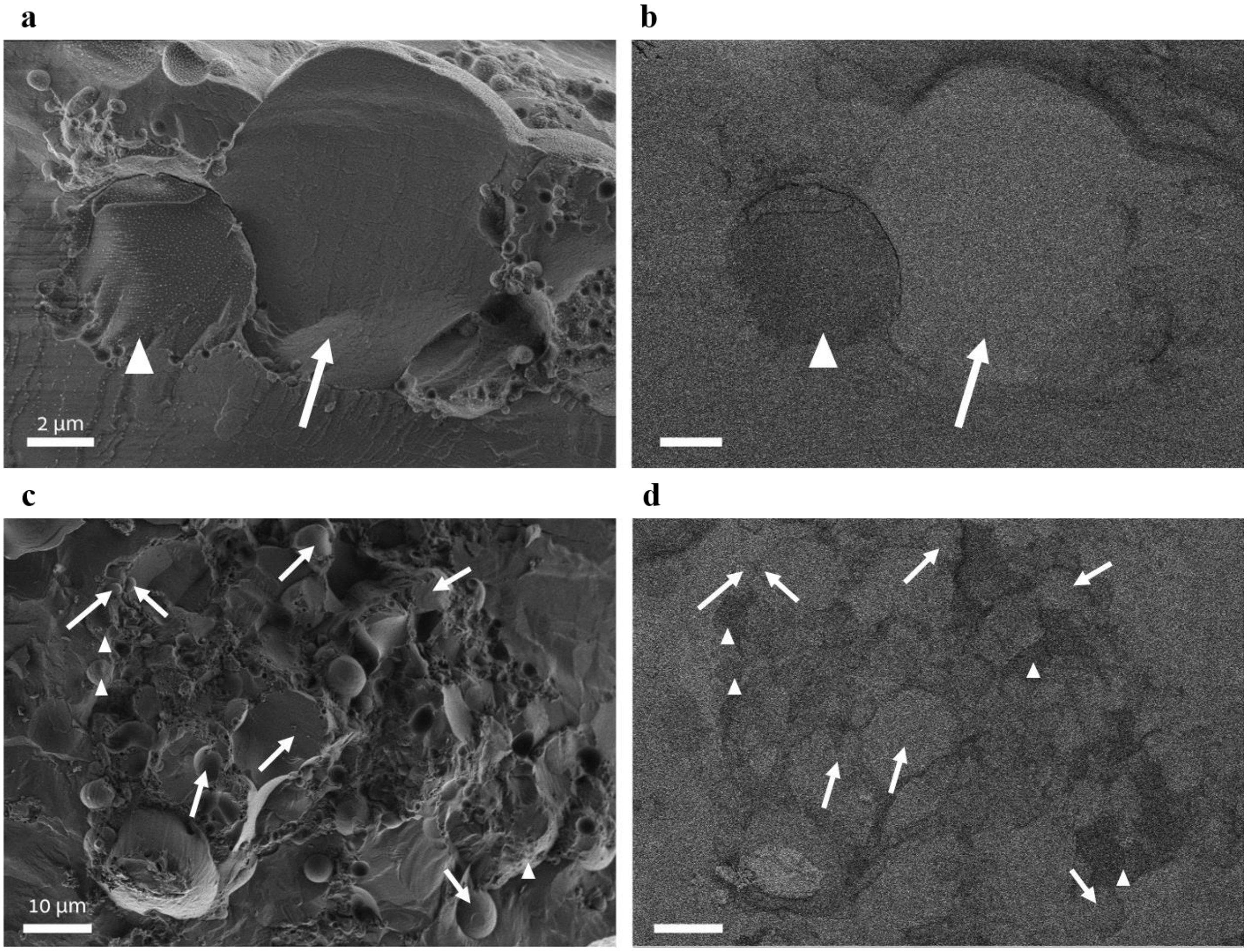

Supplementary Figure 4. The absorbance spectrum of 100 nm liposomes composed of different phospholipids. The measured absorbance spectrum of DOPC, POPC, DMPC, DPPC and HSPC liposomes between 230 and 800 nm is represented in units of wavelength (a) and photon energy (b). Data is represented as a mean (n=5 independent samples for DOPC, n=6 independent samples for POPC, n=3 independent samples for DMPC, DPPC and HSPC). Representation of the absorption in units of photon energy enabled fitting the absorption data of each phospholipid to a Lorentzian function to determine the absorption resonance of each phospholipid. (dotted line, R^2=0.9995 for DOPC, R^2=0.9993 for POPC, R^2=0.9993 for DMPC, R^2=0.9995 for DPPC, R^2=0.9996 for HSPC).

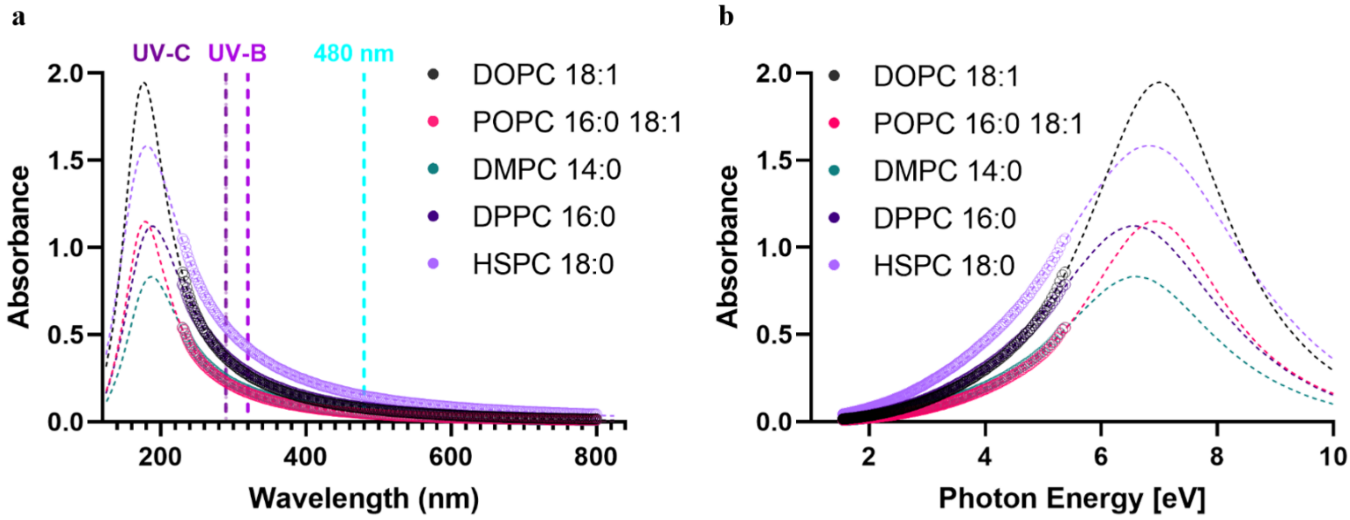

Supplementary Figure 5. The effect of different concentrations of oxidized and reduced glutathione on *Gaussia* luciferase production in cell free reactions. Data is represented as the mean ± standard deviation (n=2 independent samples).

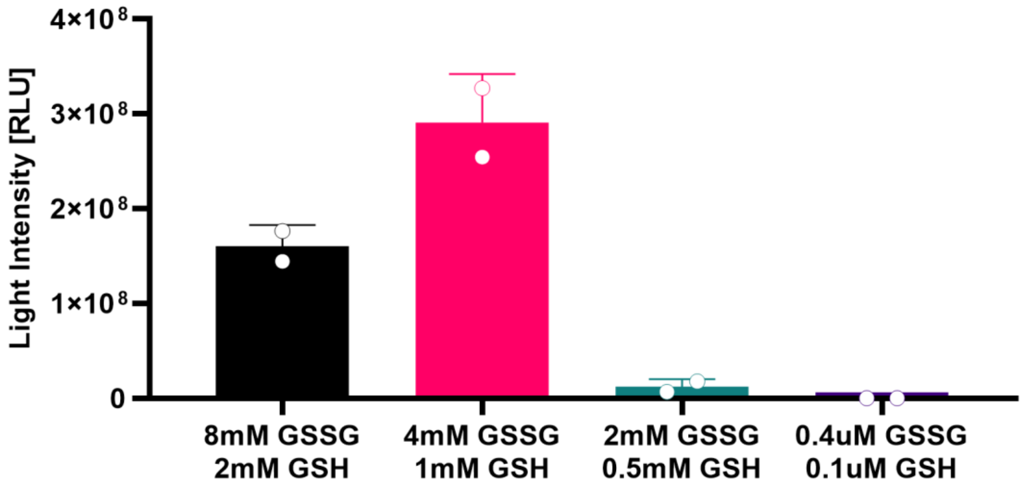

Supplementary Figure 6. SDS-Page gel with coomassie blue staining of the purified fraction of disulfide bond isomerase C (DsbC). Gel electrophoresis analysis of the purification process was performed once.

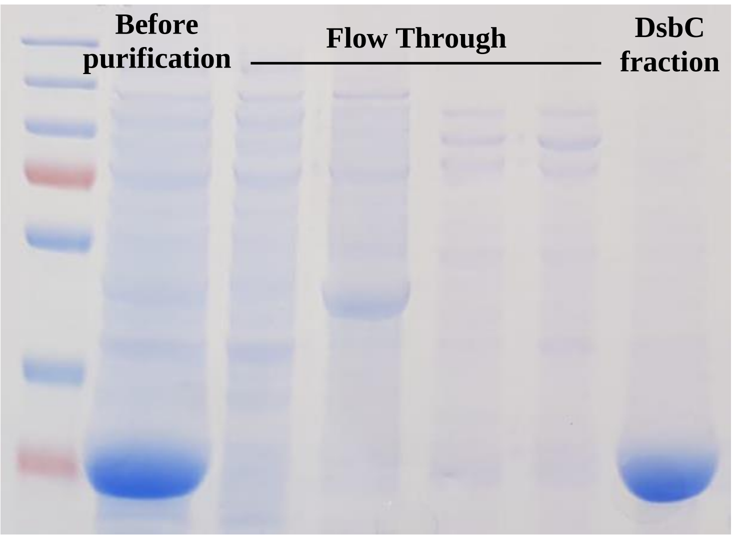

Supplementary Figure 7. Comparison of light emission levels in *Gaussia* luciferase-expressing cell-free protein expression (CFPS) reactions and synthetic cells between self-prepared and commercial internal solutions. Prior to the luminescence measurements, CFPS reactions were diluted 1000-fold and synthetic cells were all diluted to reach an absorbance of 0.1 at OD400 to ensure similar cell density. Data is represented as the mean ± standard deviation (n=2 independent samples for the commercial CFPS reactions and n=3 independent samples for all other experimental groups). Nested two-way ANOVA interaction P-value; $p=4.2\times10^{-6}$.

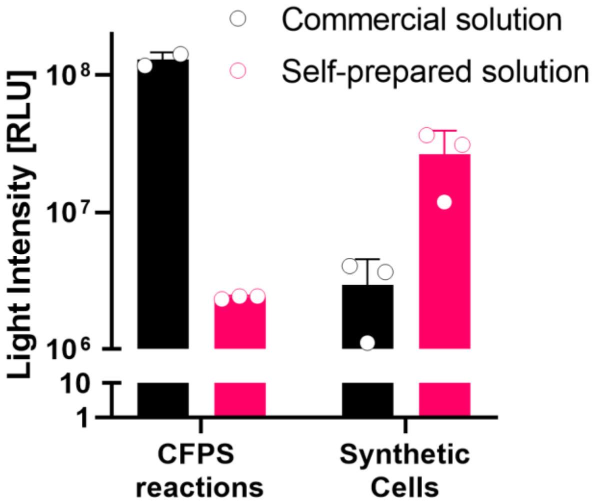

Supplementary Figure 8. The effect of incubation temperatures on *Gaussia* luciferase (Gluc) expression in SCs. Comparison of light emission from Gluc expressing SCs after incubation at 30ºC or 37ºC. Data is represented as the mean ± standard deviation (n=3 independent samples). Nested two-tailed t-test P-value; ***p*=0.0034.

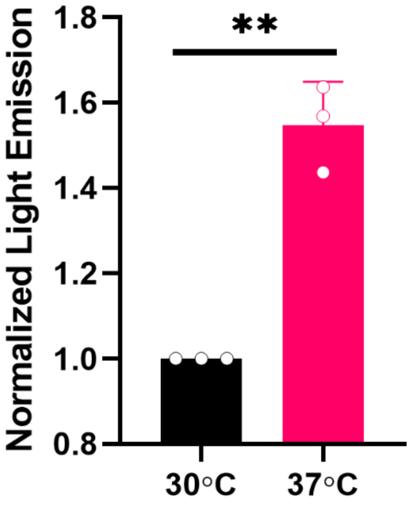

Supplementary Figure 9. Light emission from *Gaussia* luciferase-expressing synthetic cells with variable concentrations of coelenterazine ranging from 10 nM to 100 μM. Synthetic cells were diluted 400-fold prior to the measurement. Data is represented as the mean ± standard deviation (n=3 independent samples). Ordinary one-way ANOVA P-value; ***p*=0.0012.

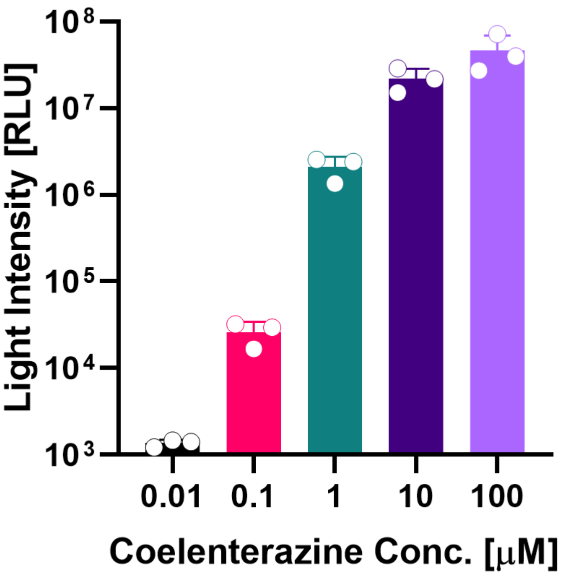

Supplementary Figure 10. Induction of photoconidiation in *Trichoderma atroviride* colonies after exposure to blue light (1-minute exposure to 15 mW cm^{-2})

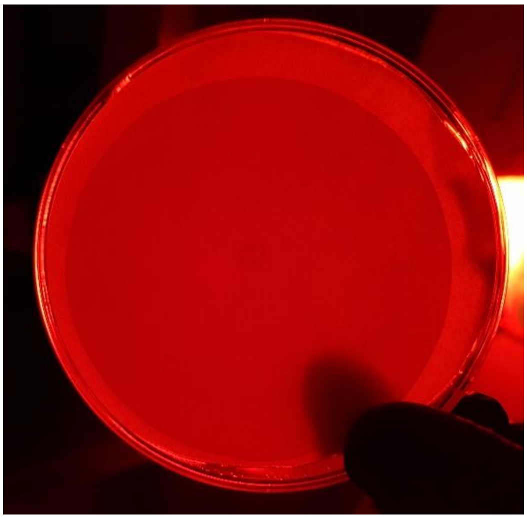

Incubated in the dark

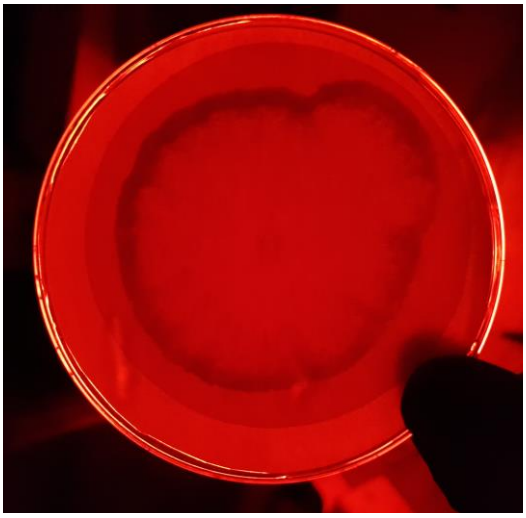

Exposed to blue light

Supplementary Figure 11. SDS-Page gel with coomassie blue staining of the purified *Gaussia* luciferase (Gluc) – EL222. Gel electrophoresis analysis of the purification process was performed once.

55kDa

35kDa

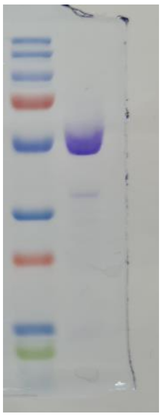

Supplementary Figure 12. The effect of coelenterazine addition in different concentrations on the yield of GFP production in cell free protein synthesis reactions. Data is represented as the mean ± standard deviation (n=2 independent samples). UPW denotes ultra-pure water.

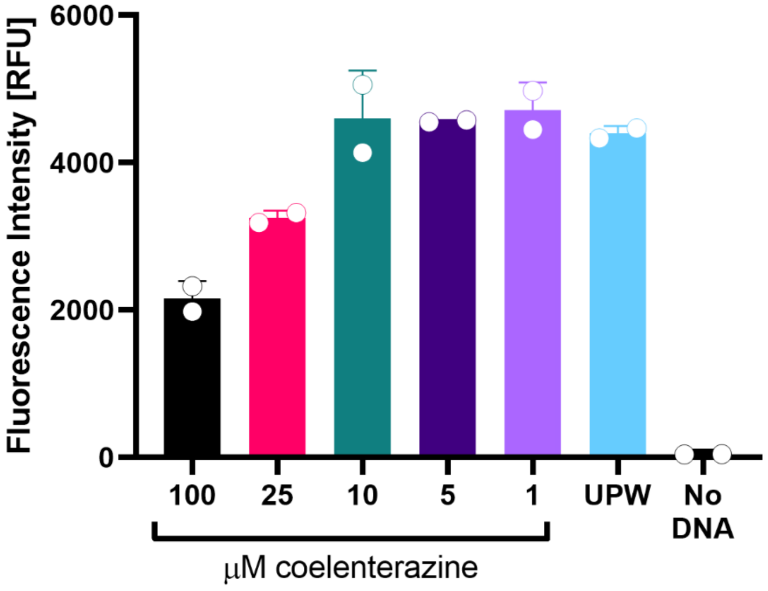

Supplementary Figure 13. SDS-Page gel with coomassie blue staining of the his-MBP-mRFP-sspB-Nano after TEV restriction, before and after purification of mRFP-sspB-Nano. Gel electrophoresis analysis of the purification process was performed once.

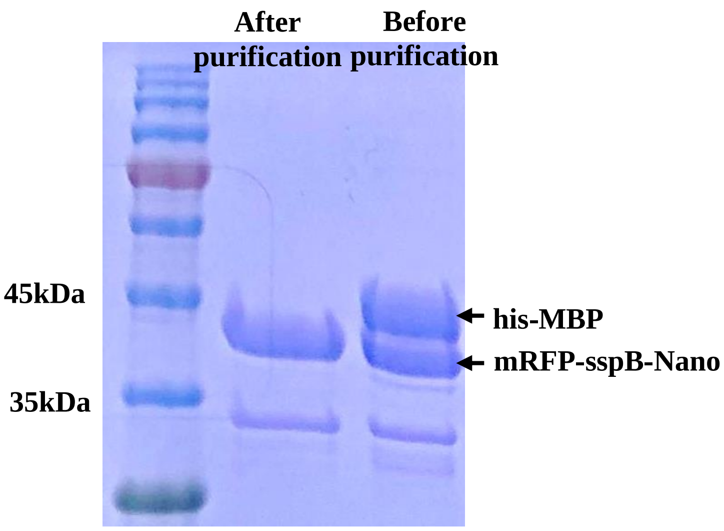

Supplementary Figure 14. Recruitment of RFP-sspB-Nano to synthetic cells with iLID functionalized membrane using a CFPS reaction expressing *Gaussia* luciferase supplemented with 100 µM coelenterazine. RFP intensity is normalized to the average intensity measured in the dark conditions. Data is represented as the mean ± s.e.m. (n=4 independent samples).

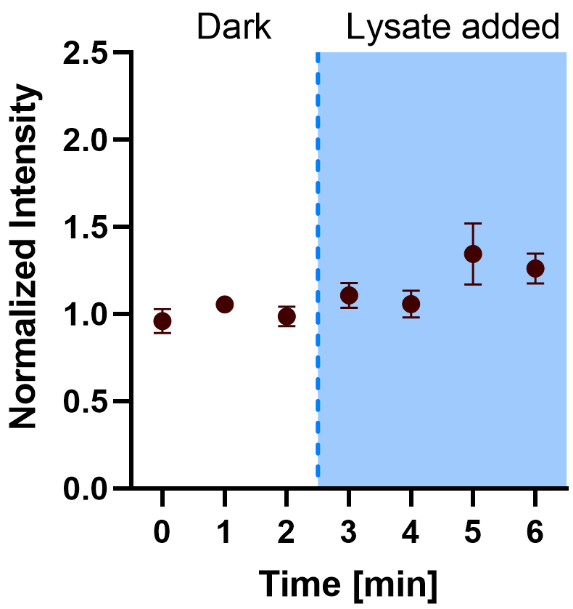

Supplementary Figure 15. SDS-Page gel with coomassie blue staining of the purified Gluc– iLID. Gel electrophoresis analysis of the purification process was performed once.

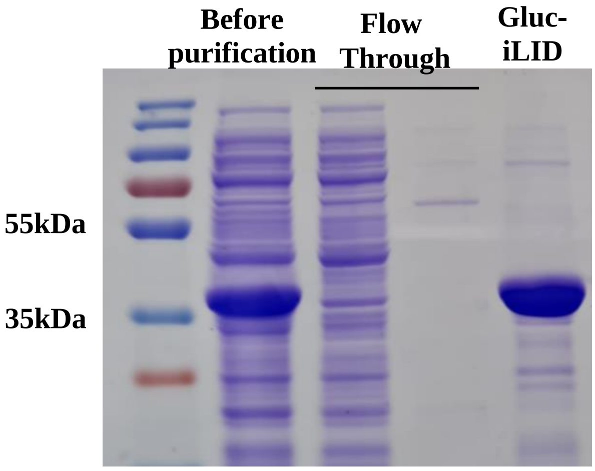

Supplementary Figure 16. Recruitment of RFP-sspB-Nano to synthetic cells functionalized with iLID or Gluc-iLID after 4 minutes of exposure to external 488 nm laser light or after four additions of 0.2 nmol of coelenterazine. Intensity was normalized to the average RFP intensity in the dark state before exposure to light or substrate. Data is represented as the mean ± standard deviation (n=3 for iLID + coelenterazine, n=11 for iLID + laser, n=14 for Gluc-iLID + coelenterazine, n=10 for gluc-iLID + laser). Student's two-tailed t-test P values; $*p = 0.0135$; $**p = 0.0092$; $****p = 3.67\times10^{-5}$.

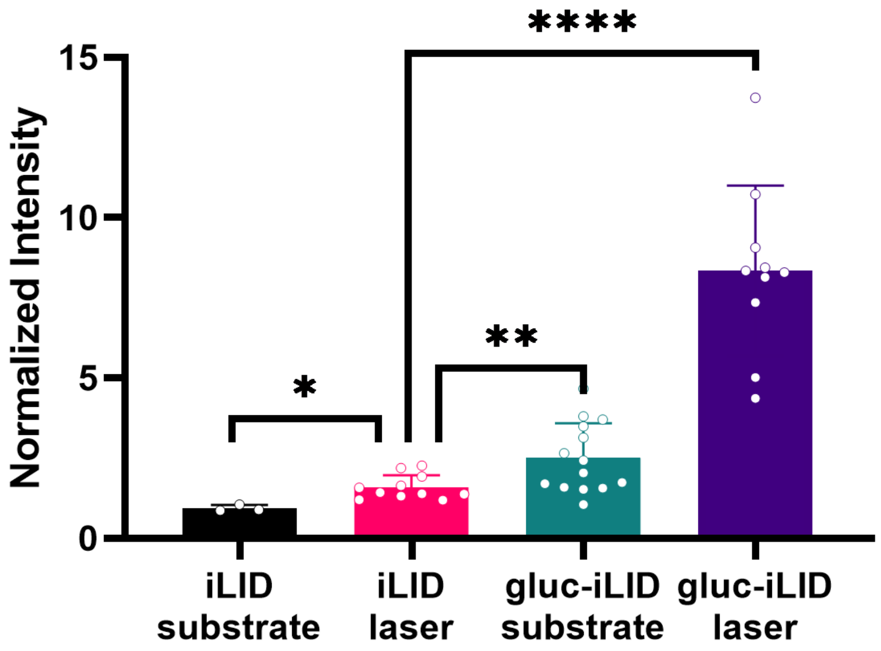

Supplement: Supplementary file 1 — Supplementary Information [file 41467_2022_29871_MOESM1_ESM.pdf]
