# Supplementary material for: Synthetic cells with self-activating optogenetic proteins communicate with natural cells
Source: Nat Commun. 2022 Apr 28;13:2328. doi: 10.1038/s41467-022-29871-8 (PMC9050678; doi:10.1038/s41467-022-29871-8)
Supplement: Supplementary file 2 — Reporting Summary [file 41467_2022_29871_MOESM2_ESM.pdf]

nature research

Corresponding author(s): Avi Schroeder

Last updated by author(s): Mar 22, 2022

Reporting Summary

Nature Research wishes to improve the reproducibility of the work that we publish. This form provides structure for consistency and transparency in reporting. For further information on Nature Research policies, see our Editorial Policies and the Editorial Policy Checklist.

Statistics

For all statistical analyses, confirm that the following items are present in the figure legend, table legend, main text, or Methods section.

n/a	Confirmed	
☐	☒	The exact sample size (*n*) for each experimental group/condition, given as a discrete number and unit of measurement
☐	☒	A statement on whether measurements were taken from distinct samples or whether the same sample was measured repeatedly
☐	☒	The statistical test(s) used AND whether they are one- or two-sided<br>*Only common tests should be described solely by name; describe more complex techniques in the Methods section.*
☒	☐	A description of all covariates tested
☐	☒	A description of any assumptions or corrections, such as tests of normality and adjustment for multiple comparisons
☐	☒	A full description of the statistical parameters including central tendency (e.g. means) or other basic estimates (e.g. regression coefficient) AND variation (e.g. standard deviation) or associated estimates of uncertainty (e.g. confidence intervals)
☐	☒	For null hypothesis testing, the test statistic (e.g. *F*, *t*, *r*) with confidence intervals, effect sizes, degrees of freedom and *P* value noted<br>*Give P values as exact values whenever suitable.*
☒	☐	For Bayesian analysis, information on the choice of priors and Markov chain Monte Carlo settings
☒	☐	For hierarchical and complex designs, identification of the appropriate level for tests and full reporting of outcomes
☒	☐	Estimates of effect sizes (e.g. Cohen's *d*, Pearson's *r*), indicating how they were calculated

Our web collection on statistics for biologists contains articles on many of the points above.

Software and code

Policy information about availability of computer code

Data collection	The i-control 1.10 software was used for collecting absorbance, luminescence and fluorescence data with the Infinite 200PRO multimode reader (TECAN). Evolution-Capt software (version 18.02) was used for collecting western blot data with the Fusion FX6 Edge. Amnis Inspire software (version 200.1.681.0) was used for collecting data in the AMNIS ImageStream®X Mk II (Luminex Corporation, USA). NIS-Elements AR (version 5.11.01) was used for collecting fluorescent microscopy data. Arduino IDE software (version 1.8.19) was used to configure the On-Off intervals in the LED illumination system.
Data analysis	Imaging data analysis was performed using Fiji Image J (version 2.0.0) and python (version 3.7). Analysis of AMNIS ImageStream data was performed using the IDEAS software (version 6.2). GraphPad Prism (version 8.3.0) was used for plot generation and statistical analysis, Matlab (version 2021b) was used for fitting a spectral line shape Lorentzian function to the absorbance data. The custom codes used in this study have been deposited in Zenodo under the following DOI: https://doi.org/10.5281/zenodo.6368147.

For manuscripts utilizing custom algorithms or software that are central to the research but not yet described in published literature, software must be made available to editors and reviewers. We strongly encourage code deposition in a community repository (e.g. GitHub). See the Nature Research guidelines for submitting code & software for further information.

Data

Policy information about availability of data

All manuscripts must include a data availability statement. This statement should provide the following information, where applicable:

- Accession codes, unique identifiers, or web links for publicly available datasets
- A list of figures that have associated raw data
- A description of any restrictions on data availability

All data supporting the findings and conclusions of this study are available within the paper, its Supplementary Information files and source data file. All other

relevant data are available from the corresponding author upon reasonable request. Source data are provided with this paper.

Field-specific reporting

Please select the one below that is the best fit for your research. If you are not sure, read the appropriate sections before making your selection.

- [x] Life sciences
- [ ] Behavioural & social sciences
- [ ] Ecological, evolutionary & environmental sciences

For a reference copy of the document with all sections, see nature.com/documents/nr-reporting-summary-flat.pdf

Life sciences study design

All studies must disclose on these points even when the disclosure is negative.

Sample size	Sample sizes were chosen based on initial pilot experiments, and at least three biological replicates were performed for most cases (which is standard in the field). The exact number of independent biological replicates are described in each figure legend.
Data exclusions	Fungi colonies plates that reached the boundaries of the plate after 3 days were excluded due to possible stress-induced conidiation. Synthetic cells with membrane oil residues were excluded prior to image acquisition (in the membrane recruitment imaging section).
Replication	The experiments shown in Fig. 2d, 3 groups in Fig. 3e, and Supplementary Fig. 5 were performed with two independent biological replicates. All other experiments were replicated successfully with at least three independent biological replicates (the number of replicates is indicated in each figure legend).
Randomization	Synthetic cell samples with identical compositions were allocated randomly to to dark / light experimental groups.
Blinding	Blinding was not applied because the features analyzed are not subject to biased interpertation (absorbance, luminescence, fluorescence intensity, etc.)

Reporting for specific materials, systems and methods

We require information from authors about some types of materials, experimental systems and methods used in many studies. Here, indicate whether each material, system or method listed is relevant to your study. If you are not sure if a list item applies to your research, read the appropriate section before selecting a response.

Materials & experimental systems

n/a	Involved in the study	
☐	☒	Antibodies
☒	☐	Eukaryotic cell lines
☒	☐	Palaeontology and archaeology
☒	☐	Animals and other organisms
☒	☐	Human research participants
☒	☐	Clinical data
☒	☐	Dual use research of concern

Methods

n/a	Involved in the study	
☒	☐	ChIP-seq
☒	☐	Flow cytometry
☒	☐	MRI-based neuroimaging

Antibodies

Antibodies used	Rabbit anti-Gaussia Luciferase, #PA1-181, ThermoFisher, Dilution 1:3750<br>Goat Anti-Rabbit IgG H&L (HRP), #ab6721, Abcam, Dilution 1:20000
Validation	Rabbit anti-Gaussia Luciferase, #PA1-181, ThermoFisher, Dilution 1:3750<br>The antibody is commercially available and was validated by the manufacturer, and in this study on purified protein.<br>https://www.thermofisher.com/antibody/product/Gaussia-luciferase-Antibody-Polyclonal/PA1-181<br><br>Goat Anti-Rabbit IgG H&L (HRP), #ab6721, Abcam, Dilution 1:20000<br>The antibody is commercially available and was validated by the manufacturer, and in previous publications<br>https://www.abcam.com/goat-rabbit-igg-hl-hrp-ab6721.html
