# Supplementary material for: Synthetic cells with self-activating optogenetic proteins communicate with natural cells
Source: Nat Commun. 2022 Apr 28;13:2328. doi: 10.1038/s41467-022-29871-8 (PMC9050678; doi:10.1038/s41467-022-29871-8)
Supplement: Supplementary file 3 — Description of Additional Supplementary Files [file 41467_2022_29871_MOESM3_ESM.pdf]

Title: Supplementary Data 1:
Description: Protein and promoter sequences.

Title: Supplementary Movie 1:
Description: Membrane-localized bioluminescent reaction generated from synthetic cells with membrane-bound Gluc-iLID proteins after addition of coelenterazine.

Title: Supplementary Movie 2:
Description: A time-lapse video of the recruitment process of RFP-sspB to Gluc-iLID labelled synthetic cells following the addition of coelenterazine.
