# Supplementary figures and images for: Synthetic cells with self-activating optogenetic proteins communicate with natural cells
Source: Nat Commun. 2022 Apr 28;13:2328. doi: 10.1038/s41467-022-29871-8 (PMC9050678; doi:10.1038/s41467-022-29871-8)

Unprocessed western blot

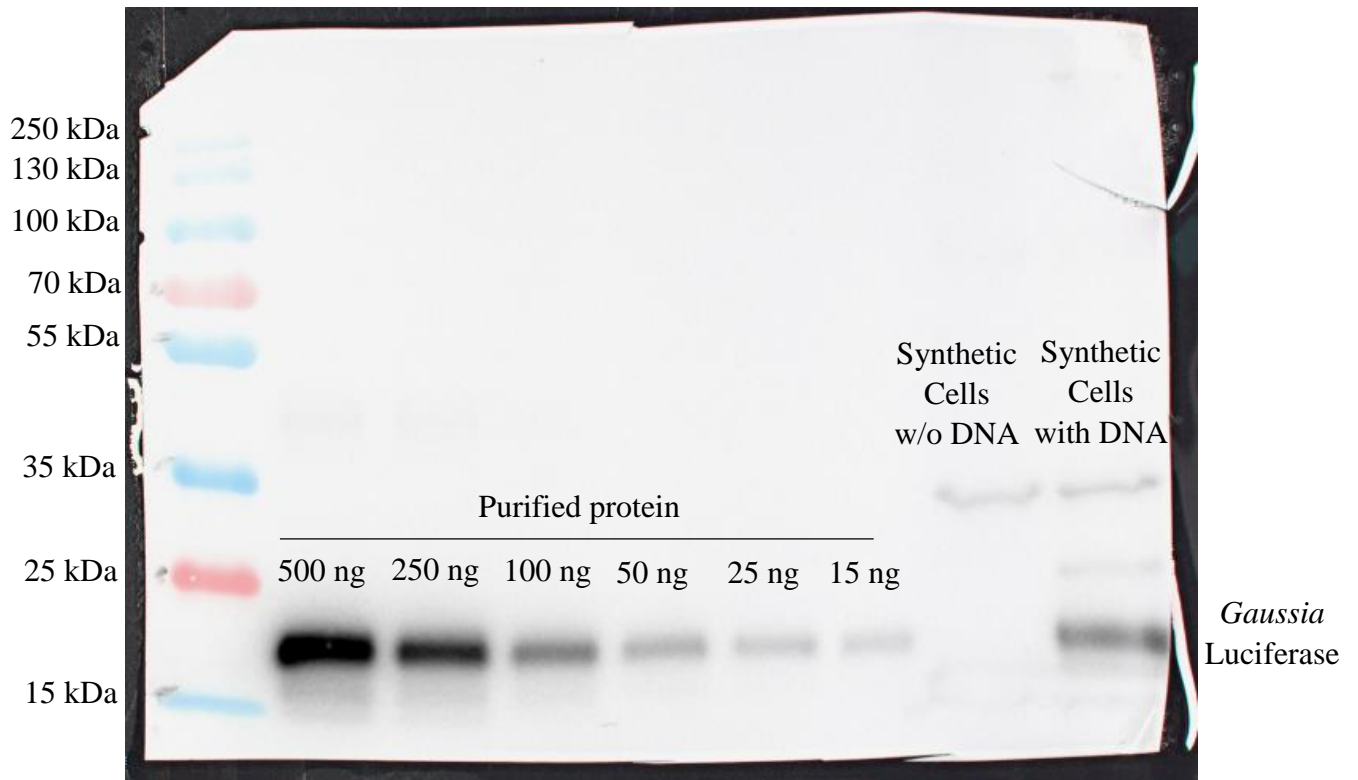

Supplement: Supplementary file 8 — Source Data [file 41467_2022_29871_MOESM8_ESM.zip › Source Data fig 2.pdf]
